# Mass and Ion Transport in Ketones and Ketone Electrolytes: Comparison with Acetate Systems

**DOI:** 10.1007/s10953-013-9983-z

**Published:** 2013-03-19

**Authors:** Dharshani N. Bopege, Matt Petrowsky, Matthew B. Johnson, Roger Frech

**Affiliations:** 1 Homer L. Dodge Department of Physics and Astronomy, University of Oklahoma, 440 W. Brooks St., Norman, OK 73019 USA; 2 Department of Chemistry and Biochemistry, University of Oklahoma, 101 Stephenson Parkway, Norman, OK 73019 USA

**Keywords:** Ionic conductivity, Diffusion coefficient, Compensated Arrhenius, Dielectric constant, Ketone electrolyte, Acetate electrolyte

## Abstract

Self-diffusion coefficient measurements were performed for pure *n*-alkyl ketone liquids using the pulsed field gradient NMR spin-echo technique. Ionic conductivities and dielectric constants of 0.0055 mol·L^−1^ tetrabutylammonium trifluoromethanesulfonate in 2-pentanone, 2-hexanone, 2-heptanone, 2-octanone, 2-nonanone, and 2-decanone were also measured. The temperature-dependent conductivities and diffusion coefficients over the range 5–80 °C can be described using the compensated Arrhenius formalism. Compensated Arrhenius equation plots were used to calculate the average activation energy for both sets of data. The average activation energy from conductivity data is approximately equal to that from diffusion data. The data for the pure ketones and ketone-based electrolytes are compared with analogous data for pure *n*-alkyl acetates and *n*-alkyl acetate-based electrolytes.

**Electronic supplementary material:**

The online version of this article (doi:10.1007/s10953-013-9983-z) contains supplementary material, which is available to authorized users.

## Introduction

Mass and charge transport in organic liquid electrolytes have garnered interest due to the use of these electrolytes in electrochemical devices. Ionic conductivities and self-diffusion coefficients are important measures of transport phenomena, although these data are conventionally described with viscosity-related models [1–3] that often predict results that do not agree with experiment [4–6]. Recently, mass and charge transport have been viewed from an entirely different perspective [7, 8], by postulating that the conductivity and diffusion coefficient assume an Arrhenius-like expression with static dielectric constant (*ε*_s_) dependence in the exponential prefactor:

1 $$ \sigma (T,\varepsilon_{\text{s}} )\,\; = \;\sigma_{0} \{ \varepsilon_{\text{s}} \left( T \right)\} { \exp }\left( { - E_{a} /RT} \right) $$

2 $$ D(T,\varepsilon_{\text{s}} ) = D_{ 0} \{ \varepsilon_{\text{s}} \left( T \right)\} { \exp }\left( { - E_{a} /RT} \right) $$

Here *σ*(*T*, *ε*_s_) is the ionic conductivity, *D*(*T*, *ε*_s_) is the self-diffusion coefficient, *σ*_0_{*ε*_s_(*T*)} and *D*_0_{*ε*_s_(*T*)} are the exponential prefactors for conductivity and diffusion, respectively. In addition, *E*_a_ is the activation energy and *T* is the temperature.

The prefactors in Eqs. 1 and 2 are temperature dependent due to the inherent temperature dependence of the dielectric constant. The dielectric constant dependence can be canceled by using a scaling procedure that has been previously described in detail [7–9]. The scaling procedure consists of dividing the temperature-dependent conductivities (or diffusion coefficients) by conductivities (or diffusion coefficients) at a reference temperature *T*_r_. The two quantities are chosen such that the temperature-dependent quantity and the reference quantity have the same value of *ε*_s_. This scaling results in compensated Arrhenius equations (CAE) for conductivity and diffusion:

3 $$ { \ln }\left( {\frac{{\sigma (T ,\varepsilon_{\text{s}} )}}{{\sigma_{\text{r}} (T_{\text{r}} ,\varepsilon_{\text{s}} )}}} \right) = - \frac{{E_{\text{a}} }}{RT} + \frac{{E_{\text{a}} }}{{RT_{\text{r}} }} $$

4 $$ { \ln }\left( {\frac{{D (T ,\varepsilon_{\text{s}} )}}{{D_{\text{r}} (T_{\text{r}} ,\varepsilon_{s} )}}} \right) = - \frac{{E_{\text{a}} }}{RT} + \frac{{E_{\text{a}} }}{{RT_{\text{r}} }} $$

The activation energies for conductivity and diffusion can be calculated from either the slope or intercept of Eqs. 3 and 4, respectively. Equations 1–4 and the postulates therein constitute the compensated Arrhenius formalism (CAF).

In this study, activation energies are reported for diffusion data of pure ketones and conductivity data of 0.0055 mol·L^−1^ tetrabutylammonium trifluoromethanesulfonate (TbaTf)–ketone solutions. We have previously reported activation energies from conductivity data for dilute ketone electrolytes that focused on short chain ketones [7]. The ketones studied here include 2-pentanone, 2-hexanone, 2-heptanone, 2-octanone, 2-nonanone and 2-decanone. This paper also compares the conductivity and diffusion data in ketones with analogous data from dilute acetate electrolytes and pure acetates [10]. Ketones and acetates are both aprotic solvents with carbonyl groups. However, the permittivity of ketones is significantly higher than that for acetates. Because the dielectric constant plays a prominent role in transport phenomena, it is important to compare conductivity and diffusion data between ketones and acetates.

## Experimental

### Materials

2-Pentanone (99+ %), 2-octanone (98 %), and 2-decanone (97 %) were obtained from Alfa Aesar, while 2-hexanone (98 % reagent grade), 2-heptanone (99 % reagent plus grade), and 2-nonanone (99+ % reagent plus grade) were from Aldrich. TbaTf (99 %) was purchased from Aldrich and used as received. The samples were prepared in a glove box under a nitrogen atmosphere (≤1 ppm H_2_O and approximate temperature 25 °C). TbaTf was dissolved in the appropriate amount of ketone to make the 0.0055 mol·L^−1^ solution, and then stirred for 24 h before use.

### Measurements

Each sample was injected into an Agilent 16452A liquid test fixture with a 2 mm spacer and immersed in an oil bath whose temperature was controlled from 5 to 85 °C, in increments of 10 °C, with a Huber Ministat 125. The conductance and capacitance were measured at each temperature (within 0.3 °C of the set temperature) over the frequency range 1 kHz to 13 MHz using a HP 4192A impedance analyzer. The conductivity, *σ*, was calculated from the measured conductance, *G*, through the equation *σ* = *L* *G* *A* ^−1^, where *L* is the electrode gap and *A* is the electrode area. The cell constant calculated from the cell geometry is 0.0176 cm^−1^. The static dielectric constant *ε*_s_ was calculated from the measured capacitance *C* using the equation *ε*_s_ = *α* *C* *C*_0_^−1^, where *α* is a variable to account for stray capacitance and *C*_0_ is the atmospheric capacitance.

A Varian VNMRS 400 MHz NMR spectrometer was utilized to measure self diffusion coefficients from 5 to 80 °C. The Larmor frequency for protons was 399.870 MHz using an Auto-X-dual broad band (5 mm) probe. The Stejskal–Tanner pulsed field gradient NMR spin-echo sequence was used for the diffusion measurements [11]. At each temperature, the gradient field strength was arrayed from 6 to 62 G·cm^−1^ and the integrated intensity of each attenuated signal was calculated. The diffusion coefficient was calculated from the slope of the plot ln(intensity) versus square of the gradient strength. The temperature was controlled using an FTS XR401 air-jet regulator.

### Data Analysis

A high degree of linearity was observed for both simple Arrhenius and compensated Arrhenius plots for the self-diffusion coefficient data of pure ketones (data are not shown). Table 1 summarizes the compensated Arrhenius *E*_a_ values at five different reference temperatures (25, 35, 45, 55, 65 °C) for pure 2-hexanone, 2-heptanone, and 2-nonanone. It is important to note that the value of *E*_a_ does not significantly depend on the choice of the reference temperature.

**Table 1:** Activation energies for pure ketones from compensated and simple Arrhenius plots of diffusion data

		CAE *E*_a_/kJ·mol^−1^		Simple Arrhenius *E*_a_/kJ·mol^−1^	
Ketone	*T*_r_ (°C)	Slope	Intercept	Ketone	Slope
2-Hexanone	25	23.8 ± 0.5	23.9 ± 0.5	2-Hexanone	15.2 ± 0.2
	35	23.7 ± 0.5	23.8 ± 0.5	2-Heptanone	15.2 ± 0.6
2-Heptanone	25	23.8 ± 0.9	24.0 ± 0.8	2-Octanone	16.1 ± 0.7
	35	23.8 ± 0.9	24.0 ± 0.9	2-Nonanone	15.1 ± 0.1
	45	23.8 ± 0.9	24.0 ± 0.9	2-Decanone	15.7 ± 0.2
	55	23.9 ± 0.9	24.0 ± 0.9		
	65	23.9 ± 0.9	23.9 ± 0.9		
2-Nonanone	65	24.0 ± 0.7	23.9 ± 0.7		

**Fig. 1:**
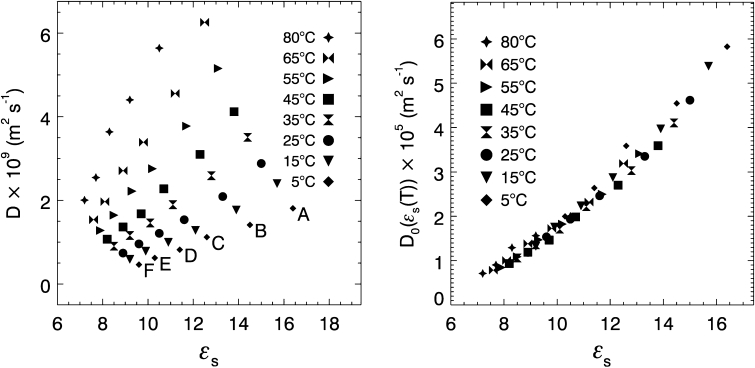
*Left*: Self-diffusion coefficients of pure ketones versus static dielectric constant from 5 to 80 °C for: (*A*) 2-pentanone, (*B*) 2-hexanone, (*C*) 2-heptanone, (*D*) 2-octanone, (*E*) 2-nonanone, and (*F*) 2-decanone. *Right*: Exponential prefactor versus dielectric constant for the diffusion data of pure ketones using *E*_a_ = 23.9 kJ·mol^−1^

The average CAE activation energy from the data in Table 1 is (23.9 ± 0.8) kJ·mol^−1^. Table 1 also lists the simple Arrhenius *E*_a_ values for each ketone obtained from a plot of ln *D* versus 1/*T*. The *E*_a_ value obtained from the simple Arrhenius plot is lower than the corresponding CAE activation energies, which is a trend that has been observed in other systems [8–10]. Figure 1a plots self-diffusion coefficients versus static dielectric constant for pure ketones. Six well-separated curves are observed, one each for the temperature dependent data of each ketone.

The exponential prefactors, *D*_0_, were calculated from Eq. 2 by dividing the temperature dependent diffusion coefficients by the Boltzmann factor, exp(–*E*_a_/*RT*). The plot of exponential prefactor versus dielectric constant yields a single master curve in Fig. 1b. This single master curve can be observed only for a narrow range of *E*_a_ values (22.5–25.5 kJ·mol^−1^). The CAE gives *E*_a_ values that are within this range, while the simple Arrhenius plots yield activation energies that do not result in a master curve.

For conductivity data of 0.0055 mol·L^−1^ TbaTf–2-ketone systems, CAE plots exhibit linear behavior while simple Arrhenius plots are approximately linear but do show slight curvature (data not shown). The resulting CAE and simple Arrhenius *E*_a_ values are reported in Table 2. Similar to the diffusion data, simple Arrhenius *E*_a_ values are lower than those from the CAE. The average CAE activation energy from the data in Table 2 is (24.1 ± 0.8) kJ·mol^−1^; this value was utilized to determine the conductivity exponential prefactors by dividing the temperature-dependent conductivities in Eq. 1 by the Boltzmann factor.

**Table 2:** Activation energies from compensated and simple Arrhenius plots resulting from conductivity data for 0.0055 mol·L^−1^ TbaTf–2-ketones

		CAE *E*_a_/kJ·mol^−1^		Simple Arrhenius *E*_a_/kJ·mol^−1^	
Ketone	*T*_r_ (°C)	Slope	Intercept	Ketone	Slope
2-Hexanone	25	23.1 ± 0.6	23.2 ± 0.6	2-Pentanone	5.0 ± 0.1
	35	22.7 ± 0.5	22.8 ± 0.5	2-Hexanone	5.2 ± 0.1
2-Heptanone	25	23.9 ± 1.0	24.1 ± 1.0	2-Heptanone	5.6 ± 0.2
	35	24.1 ± 0.9	24.3 ± 0.9	2-Octanone	6.3 ± 0.2
	45	23.4 ± 0.8	23.6 ± 0.8	2-Nonanone	7.2 ± 0.2
	55	23.0 ± 0.5	23.2 ± 0.6	2-Decanone	8.5 ± 0.2
2-Octanone	45	25 ± 1	25 ± 1		
	55	25.3 ± 0.9	25.3 ± 0.9		
	65	24.8 ± 0.9	24.7 ± 0.9		
2-Nonanone	80	25.4 ± 0.8	25.1 ± 0.9		

**Fig. 2:**
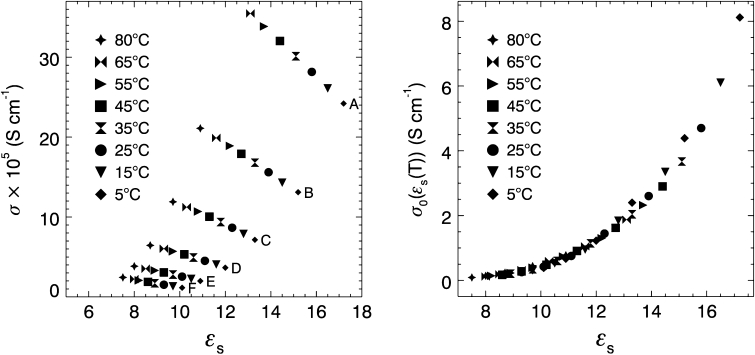
*Left*: Conductivity versus static dielectric constant for 0.0055 mol·L^−1^ TbaTf–ketone solutions of (*A*) 2-pentanone, (*B*) 2-hexanone, (*C*) 2-heptanone, (*D*) 2-octanone, (*E*) 2-nonanone, and (*F*) 2-decanone over the temperature range 5 to 80 °C. *Right*: Exponential prefactor versus the dielectric constant for 0.0055 mol·L^−1^ TbaTf–ketone solutions using *E*_a_ = 24.1 kJ·mol^−1^

Temperature-dependent ionic conductivities are plotted against temperature-dependent dielectric constants in Fig. 2a for the 0.0055 mol·L^−1^ TbaTf–ketone data. Six distinct curves are observed, one for each ketone electrolyte solution. However, a single master curve is observed when the exponential prefactors are plotted against the static dielectric constant, as shown in Fig. 2b. A master curve is only observed for *E*_a_ values in the narrow range from 22 to 27 kJ·mol^−1^. Similar to the diffusion results, the CAE activation energies result in a master curve, while those from the simple Arrhenius equation do not.

**Fig. 3:**
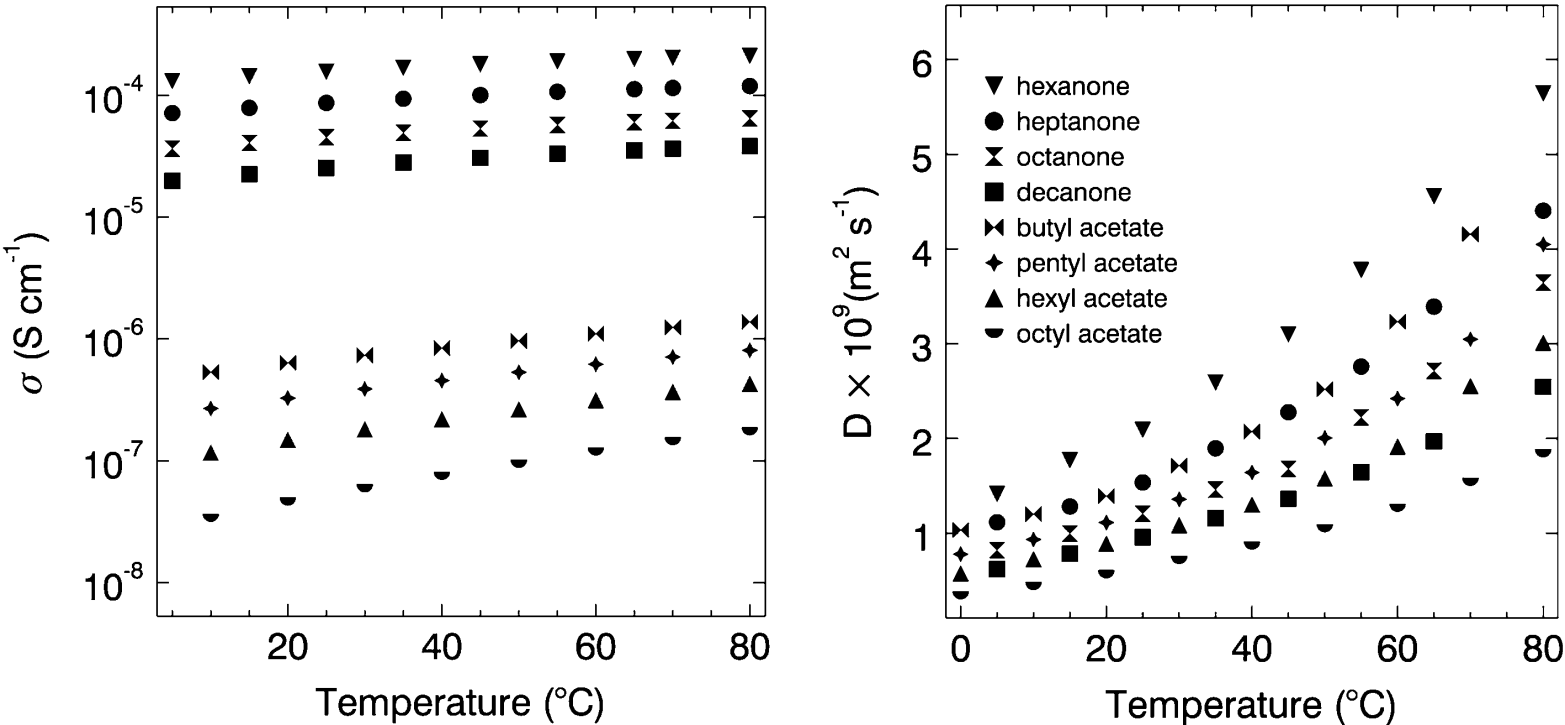
*Left*: Conductivity versus temperature for 0.0055 mol·L^−1^ TbaTf–2-ketone solutions and 0.0055 mol·L^−1^ TbaTf–*n*-acetate solutions from 5 to 80 °C. *Right*: Self-diffusion coefficient versus temperature for pure ketone and pure acetate solvents

A comparison of the conductivities of the 0.0055 mol·L^−1^ TbaTf–ketone solutions in the present study with the conductivities of 0.0055 mol·L^−1^ TbaTf–acetate solutions from an earlier study [10] is shown in Fig. 3a. The conductivities of both ketone and acetate solutions increase with increasing temperature and decreasing alkyl chain length as expected. However, despite sharing commonalities in their chemical structures (the carbonyl oxygen common to both presumably coordinates the cation in electrolyte solutions), the conductivities of the TbaTf–ketone solutions are significantly higher than those of the TbaTf–acetate solutions. What is the origin of this difference?

**Fig. 4:**
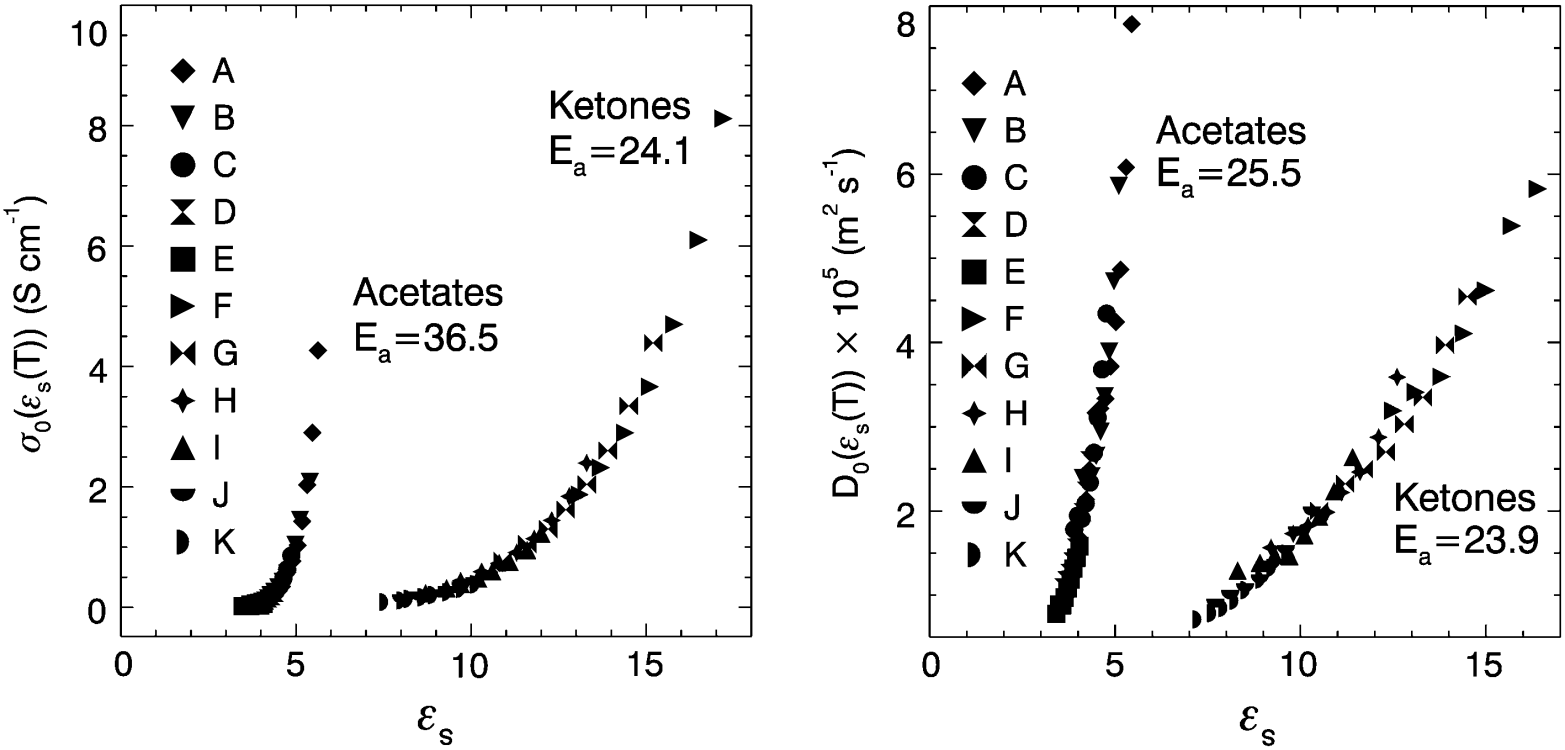
*Left*: Exponential prefactor versus dielectric constant for the conductivity data of 0.0055 mol·L^−1^ TbaTf-acetates and 0.0055 mol·L^−1^ TbaTf-ketones. *Right*: Exponential prefactor versus dielectric constant for the diffusion data of pure acetates and pure ketones. The solvents are designated by: (*A*) butyl acetate, (*B*) pentyl acetate, (*C*) hexyl acetate, (*D*) octyl acetate, (*E*) decyl acetate, (*F*) 2-pentanone, (*G*) 2-hexanone, (*H*) 2-heptanone, (*I*) 2-octanone, (*J*) 2-nonanone, (*K*) 2-decanone. The units of *E*_a_ are kJ·mol^−1^

The CAF provides important insight into this difference. In Eq. 1 there are two factors that control the conductivity: the exponential prefactor *σ*_0_ and the Boltzmann factor exp(–*E*_a_/*RT)*. Figure 4a shows that the exponential prefactors calculated from the conductivity data are very similar for the acetates and ketones. By way of comparison, a 0.004 mol·L^−1^ solution of TbaTf in 1-hexanol has *σ*_0_ = 1.10 × 10^4^ S·cm^−1^ at 5 °C (*ε*_s_ = 15.5) and *σ*_0_ = 2.70 × 10^2^ S·cm^−1^ at 85 °C (*ε*_s_ = 8.0) (unpublished data).

In a previous study, we found that the average CAE conductivity activation energy for a series of 0.0055 mol·L^−1^ TbaTf–acetate solutions was 36.5 kJ·mol^−1^, which is significantly higher than the value of 24.1 kJ·mol^−1^ for the 0.0055 mol·L^−1^ TbaTf–ketone solutions in the present study. Since the values of the prefactors are so similar, the higher conductivities of the ketone electrolytes originate in their markedly lower activation energies compared with the acetate electrolytes.

The conductivity of an electrolyte can be expressed as *σ* = *Σ*_*i*_ *n*_*i*_ *q*_*i*_ *μ*_*i*_, where the summation runs over all charged species in the system and *n*_*i*_ is the number density of charge carriers of type *i* with charge *q*_*i*_ and mobility *μ*_*i*_. The conductivity difference between the ketone and acetate solutions cannot be due to the number density of ions since both electrolytes have the same salt concentration (0.0055 mol·L^−1^). Numerous studies of ionic association in various Tba salt solutions have shown that weak, solvent-separated ion pairing occurs [12–15]. However, the charge-protected Tba cation does not form discrete, spectroscopically observable ionic species such as are found in lithium and sodium salt solutions [16–18]. Consequently, TbaTf electrolytes consist only of spectroscopically “free” ions, and ion mobility must be the key factor that controls the conductivity in these electrolytes. Accordingly, we conclude that the ion mobilities in the ketone electrolytes are substantially higher than those in the acetate electrolytes.

In contrast to the conductivity data, diffusion coefficients are comparable between pure ketones and acetates for similar temperatures and chain lengths as shown in Fig. 3b. The average compensated Arrhenius diffusion activation energy for the pure ketones is 23.9 kJ·mol^−1^ and that for the pure acetates is 25.5 kJ·mol^−1^. The diffusion prefactors are also comparable between acetates and ketones as seen in Fig. 4b; therefore, it is not surprising that the values of the diffusion coefficients of the ketones and acetates are similar. Indeed the primary difference between these two solvent families is that the acetate prefactor master curve is horizontally shifted on the permittivity axis from the ketone curve for both conductivity and diffusion prefactors in Fig. 4. This shift is due to the difference in permittivity originating in the difference in dipole moments for these two solvent families. For example, the gas phase permanent moment of ethyl acetate (C_4_H_8_O_2_) is 1.78 D [19] while that of 2-butanone (C_4_H_8_O) is 2.78 D [20].

The *E*_a_ for diffusion in pure ketones is approximately equivalent to the *E*_a_ for conductivity in the dilute TbaTf–ketone solutions as indicated in Fig. 4. However, the addition of a small amount of TbaTf to a pure acetate solvent increases the average activation energy from 25.5 to 36.5 kJ mol^−1^ in the 0.0055 mol·L^−1^ solution. This difference may originate in the lower permittivities of the acetate systems that lead to stronger ion–solvent interactions and contribute in large part to the 10 kJ·mol^−1^ difference in their *E*_a_ values.

One of our previous CAF studies involved 0.0055 mol·L^−1^ TbaTf–ketone solution conductivity data that included the short chain ketones 2-butanone and acetone. This study resulted in an average activation energy of ~16 kJ·mol^−1^ [7]. Numerous CAF studies of other solvent-based systems (alcohols, ketones, acetates, nitriles, acyclic carbonates) have led to the conclusion that including the shortest alkyl chain members of a solvent family in the CAF analysis produces activation energies that are somewhat lower than if these members are omitted. The present work gives an average conductivity activation energy of 24.1 kJ·mol^−1^ by leaving out the acetone and 2-butanone data. Further, the activation energies calculated here for 2-hexanone, 2-heptanone, 2-octanone, and 2-nonanone are remarkably close to each other as seen in Table 2.

## Electronic supplementary material

Below is the link to the electronic supplementary material.

Supplementary material 1 (DOCX 29 kb)
