# Supplementary material for: Mass and Ion Transport in Ketones and Ketone Electrolytes: Comparison with Acetate Systems
Source: J Solution Chem. 2013 Mar 19;42(3):584–91. doi: 10.1007/s10953-013-9983-z (PMC3610029; doi:10.1007/s10953-013-9983-z)
Supplement: Supplementary file 1 — Supplementary material 1 (DOCX 29 kb) [file 10953_2013_9983_MOESM1_ESM.docx]

**Journal of Solution Chemistry electronic supplementary material**

**Mass and Ion Transport in Ketones and Ketone Electrolytes: Comparison with Acetate Systems**

Dharshani N. Bopege,^†^ Matt Petrowsky,^‡^ Matthew B.

Johnson,^†^ and Roger Frech*^∗^*^,‡^

† Homer L. Dodge Department of Physics and Astronomy, University of Oklahoma, 440 W. Brooks St., Norman, OK 73019, USA

‡Department of Chemistry and Biochemistry, University of

Oklahoma, 101 Stephenson Parkway, Norman, OK 73019, USA

e-mail: [rfrech@ou.edu](mailto:rfrech@ou.edu); telephone: +1 (405)325 3831; fax: +1 (405)325 6111

The following tables give ionic conductivity (*σ*) and static dielectric constant (*ε*_s_) data for 0.0055 mol·L^–1^ TbaTf–solvent solutions, where TbaTf is an abbreviation for tetrabutylammonium trifluoromethanesulfonate. Additionally, self-diffusion coefficients (*D*) and static dielectric constants are given for the pure solvents. Finally, density data are given for the pure solvents.

2-ketone data

| Temperature  (°C) | *σ*  (S·cm^–1^) /  0.0055 mol·L^–1^  TbaTf | *ε*_s_ /  0.0055 mol·L^–1^ TbaTf | *D*  (m^2^·s^–1^)  pure | *ε*_s_  pure | Density  (g·cm^–3^)  pure |
| --- | --- | --- | --- | --- | --- |
| 2-pentanone | | | | | |
| 5 | 2.41 × 10^–4^ | 17.2 | 1.86 × 10^–9^ | 16.4 | 0.8208 |
| 15 | 2.60 × 10^–4^ | 16.5 | 2.38 × 10^–9^ | 15.7 | 0.8113 |
| 25 | 2.81 × 10^–4^ | 15.8 | 2.97 × 10^–9^ | 15.0 | 0.8016 |
| 35 | 3.01 × 10^–4^ | 15.1 | 3.59 × 10^–9^ | 14.4 | 0.7918 |
| 45 | 3.20 × 10^–4^ | 14.4 | 4.32 × 10^–9^ | 13.8 | 0.7819 |
| 55 | 3.38 × 10^–4^ | 13.8 | 5.40 × 10^–9^ | 13.1 | 0.7719 |
| 65 | 3.54 × 10^–4^ | 13.1 | 6.26 × 10^–9^ | 12.5 | 0.7618 |
| 2-hexanone | | | | | |
| 5 | 1.30 × 10^–4^ | 15.2 | 1.41 × 10^–9^ | 14.6 | 0.8250 |
| 15 | 1.43 × 10^–4^ | 14.6 | 1.76 × 10^–9^ | 13.9 | 0.8161 |
| 25 | 1.56 × 10^–4^ | 13.9 | 2.06 × 10^–9^ | 13.3 | 0.8071 |
| 35 | 1.67 × 10^–4^ | 13.4 | 2.56 × 10^–9^ | 12.8 | 0.7980 |
| 45 | 1.79 × 10^–4^ | 12.8 | 3.04 × 10^–9^ | 12.3 | 0.7888 |
| 55 | 1.89 × 10^–4^ | 12.2 | 3.67 × 10^–9^ | 11.7 | 0.7795 |
| 65 | 1.98 × 10^–4^ | 11.6 | 4.40 × 10^–9^ | 11.2 | 0.7702 |
| 80 | 2.10 × 10^–4^ | 10.9 | 5.52 × 10^–-9^ | 10.5 | 0.7560 |
| 2-heptanone | | | | | |
| 5 | 7.13 × 10^–5^ | 13.3 | 1.06 × 10^–9^ | 12.6 | 0.8283 |
| 15 | 7.87 × 10^–5^ | 12.8 | 1.35 × 10^–9^ | 12.1 | 0.8198 |
| 25 | 8.64 × 10^–5^ | 12.3 | 1.61 × 10^–9^ | 11.6 | 0.8112 |
| 35 | 9.35 × 10^–5^ | 11.8 | 2.00 × 10^–9^ | 11.2 | 0.8026 |
| 45 | 1.00 × 10^–4^ | 11.3 | 2.42 × 10^–9^ | 10.7 | 0.7939 |
| 55 | 1.07 × 10^–4^ | 10.8 | 3.02 × 10^–9^ | 10.2 | 0.7851 |
| 65 | 1.12 × 10^–4^ | 10.3 | 3.74 × 10^–-9^ | 9.80 | 0.7763 |
| 80 | 1.19 × 10^–4^ | 9.67 | 4.71 × 10^–-9^ | 9.18 | 0.7629 |
| 2-octanone | | | | | |
| 5 | 3.64 × 10^–5^ | 12.0 | 8.21 × 10^–10^ | 11.4 | 0.8308 |
| 15 | 4.06 × 10^–5^ | 11.5 | 9.77 × 10^–10^ | 10.9 | 0.8226 |
| 25 | 4.50 × 10^–5^ | 11.1 | 1.18 × 10^–9^ | 10.5 | 0.8143 |
| 35 | 4.92 × 10^–5^ | 10.6 | 1.42 × 10^–9^ | 10.1 | 0.8061 |
| 45 | 5.32 × 10^–5^ | 10.2 | 1.65 × 10^–9^ | 9.70 | 0.7977 |
| 55 | 5.69 × 10^–5^ | 9.71 | 2.21 × 10^–9^ | 9.28 | 0.7893 |
| 65 | 6.02 × 10^–5^ | 9.30 | 2.69 × 10^–9^ | 8.89 | 0.7809 |
| 80 | 6.43 × 10^–5^ | 8.74 | 3.55 × 10^–9^ | 8.35 | 0.7681 |
| 2-nonanone | | | | | |
| 5 | 1.98 × 10^–5^ | 10.9 | 6.18 × 10^–10^ | 10.4 | 0.8338 |
| 15 | 2.24 × 10^–5^ | 10.5 | 7.74 × 10^–10^ | 9.96 | 0.8258 |
| 25 | 2.53 × 10^–5^ | 10.1 | 9.45 × 10^–10^ | 9.57 | 0.8178 |
| 35 | 2.80 × 10^–5^ | 9.70 | 1.14 × 10^–9^ | 9.22 | 0.8098 |
| 45 | 3.06 × 10^–5^ | 9.26 | 1.34 × 10^–9^ | 8.88 | 0.8017 |
| 55 | 3.30 × 10^–5^ | 8.87 | 1.62 × 10^–9^ | 8.51 | 0.7936 |
| 65 | 3.52 × 10^–5^ | 8.55 | 1.94 × 10^–9^ | 8.16 | 0.7855 |
| 80 | 3.83 × 10^–5^ | 8.05 | 2.51 × 10^–9^ | 7.68 | 0.7731 |
| 2-decanone | | | | | |
| 5 | 1.14 × 10^–5^ | 10.1 | 4.64 × 10^–10^ | 9.60 | 0.8354 |
| 15 | 1.32 × 10^–5^ | 9.70 | 5.97 × 10^–10^ | 9.22 | 0.8277 |
| 25 | 1.52 × 10^–5^ | 9.30 | 7.47 × 10^–10^ | 8.88 | 0.8199 |
| 35 | 1.70 × 10^–5^ | 8.93 | 9.10 × 10^–10^ | 8.55 | 0.8120 |
| 45 | 1.88 × 10^–5^ | 8.57 | 1.08 × 10^–9^ | 8.24 | 0.8042 |
| 55 | 2.06 × 10^–5^ | 8.22 | 1.29 × 10^–9^ | 7.91 | 0.7963 |
| 65 | 2.22 × 10^–5^ | 7.89 | 1.54 × 10^–9^ | 7.60 | 0.7883 |
| 80 | 2.44 × 10^–5^ | 7.44 | 1.98 × 10^–9^ | 7.17 | 0.7764 |

*n*-acetate data

| Temperature  (^o^C) | *σ*  (S·cm^–1^) /  0.0055 mol·L^–1^  TbaTf | *ε*_s_  0.0055 mol·L^–1^  TbaTf | *D*  (m^2^·s^–1^)  pure | *ε*_s_  pure | Density  (g·cm^–3^)  pure |
| --- | --- | --- | --- | --- | --- |
| *n*-butyl acetate | | | | | |
| 0 | 4.45 × 10^–7^ | 5.64 | 1.03 × 10^–9^ | 5.46 |  |
| 10 | 5.35 × 10^–7^ | 5.47 | 1.20 × 10^–9^ | 5.31 | 0.8915 |
| 20 | 6.36 × 10^–7^ | 5.33 | 1.39 × 10^–9^ | 5.16 | 0.8813 |
| 30 | 7.34 × 10^–7^ | 5.18 | 1.71 × 10^–9^ | 5.03 | 0.8710 |
| 40 | 8.41 × 10^–7^ | 5.04 | 2.07 × 10^–9^ | 4.90 | 0.8606 |
| 50 | 9.65 × 10^–7^ | 4.89 | 2.52 × 10^–9^ | 4.75 | 0.8501 |
| 60 | 1.10 × 10^–6^ | 4.75 | 3.23 × 10^–9^ | 4.61 | 0.8395 |
| 70 | 1.24 × 10^–6^ | 4.62 | 4.16 × 10^–9^ | 4.48 | 0.8288 |
| 80 | 1.37 × 10^–6^ | 4.49 |  |  |  |
| *n*-pentyl acetate | | | | | |
| 0 | 2.18 × 10^–7^ | 5.26 | 7.79 × 10^–10^ | 5.11 |  |
| 10 | 2.68 × 10^–7^ | 5.12 | 9.34 × 10^–10^ | 4.98 | 0.8862 |
| 20 | 3.27 × 10^–7^ | 4.99 | 1.11 × 10^–9^ | 4.85 | 0.8766 |
| 30 | 3.89 × 10^–7^ | 4.87 | 1.36 × 10^–9^ | 4.73 | 0.8669 |
| 40 | 4.55 × 10^–7^ | 4.75 | 1.64 × 10^–9^ | 4.61 | 0.8571 |
| 50 | 5.32 × 10^–7^ | 4.62 | 2.01 × 10^–9^ | 4.49 | 0.8472 |
| 60 | 6.20 × 10^–7^ | 4.50 | 2.42 × 10^–9^ | 4.36 | 0.8373 |
| 70 | 7.11 × 10^–7^ | 4.38 | 3.05 × 10^–9^ | 4.25 | 0.8272 |
| 80 | 8.06 × 10^–7^ | 4.27 | 4.05 × 10^–9^ | 4.13 | 0.8171 |
| *n*-hexyl acetate | | | | | |
| 0 | 9.01 × 10^–8^ | 4.88 | 5.77 × 10^–10^ | 4.78 |  |
| 10 | 1.17 × 10^–7^ | 4.76 | 7.27 × 10^–10^ | 4.66 | 0.8818 |
| 20 | 1.49 × 10^–7^ | 4.65 | 8.89 × 10^–10^ | 4.54 | 0.8727 |
| 30 | 1.81 × 10^–7^ | 4.54 | 1.09 × 10^–9^ | 4.43 | 0.8635 |
| 40 | 2.19 × 10^–7^ | 4.44 | 1.30 × 10^–9^ | 4.33 | 0.8542 |
| 50 | 2.64 × 10^–7^ | 4.32 | 1.58 × 10^–9^ | 4.21 | 0.8449 |
| 60 | 3.14 × 10^–7^ | 4.22 | 1.92 × 10^–9^ | 4.11 | 0.8355 |
| 70 | 3.67 × 10^–7^ | 4.11 | 2.55 × 10^–9^ | 4.01 | 0.8261 |
| 80 | 4.28 × 10^–7^ | 4.01 | 3.01 × 10^–9^ | 3.91 | 0.8165 |
| *n*-octyl acetate | | | | | |
| 0 | 2.49 × 10^–8^ | 4.40 | 3.45 × 10^–10^ | 4.32 |  |
| 10 | 3.39 × 10^–8^ | 4.31 | 4.43 × 10^–10^ | 4.22 | 0.8767 |
| 20 | 4.61 × 10^–8^ | 4.21 | 5.68 × 10^–10^ | 4.13 | 0.8682 |
| 30 | 5.94 × 10^–8^ | 4.13 | 7.18 × 10^–10^ | 4.04 | 0.8597 |
| 40 | 7.53 × 10^–8^ | 4.04 | 8.71 × 10^–10^ | 3.96 | 0.8511 |
| 50 | 9.43 × 10^–8^ | 3.95 | 1.05 × 10^–9^ | 3.86 | 0.8425 |
| 60 | 1.19 × 10^–7^ | 3.86 | 1.27 × 10^–9^ | 3.78 | 0.8339 |
| 70 | 1.45 × 10^–7^ | 3.78 | 1.54 × 10^–9^ | 3.69 | 0.8252 |
| 80 | 1.74 × 10^–7^ | 3.70 | 1.84 × 10^–9^ | 3.61 | 0.8165 |
| *n*-decyl acetate | | | | | |
| 0 | 5.88 × 10^–9^ | 4.08 | 2.10 × 10^–10^ | 4.05 |  |
| 10 | 6.35 × 10^–9^ | 3.99 | 2.85 × 10^–10^ | 3.96 | 0.8740 |
| 20 | 9.01 × 10^–9^ | 3.91 | 3.76 × 10^–10^ | 3.88 | 0.8659 |
| 30 | 1.68 × 10^–8^ | 3.84 | 4.77 × 10^–10^ | 3.80 | 0.8578 |
| 40 | 2.49 × 10^–8^ | 3.77 | 6.02 × 10^–10^ | 3.73 | 0.8497 |
| 50 | 3.31 × 10^–8^ | 3.70 | 7.33 × 10^–10^ | 3.65 | 0.8416 |
| 60 | 4.63 × 10^–8^ | 3.62 | 8.82 × 10^–10^ | 3.57 | 0.8335 |
| 70 | 6.02 × 10^–8^ | 3.55 | 1.17 × 10^–9^ | 3.50 | 0.8253 |
| 80 | 7.50 × 10^–8^ | 3.48 | 1.31 × 10^–9^ | 3.43 | 0.8171 |
